# Meetings of Societies

**Published:** 1933

**Authors:** 


### Meetings of Societies.

### Bristol Medico-Chirurgical Society.

The sixth meeting of the session was held in the Physiological Lecture Theatre of the University on Wednesday, 8th March, at 8.30 p.m.

Dr. G. B. Bush and Dr. A. L. Taylor showed X-rays and pathological specimens.

Dr. A. L. Taylor described one of the specimens and Mr. Wright spoke of the clinical history of the case.

Mr. E. Watson-Williams showed two cases of œsophageal obstruction and Mr. Wright discussed the diagnosis in one of them.

Mr. Drew Smythe then read a paper on the "**B Coli Infections of the Female Urinary Tract.**"

A discussion followed in which the President, Professor Statham, Dr. Hector, Mr. Walters, and Dr. Frank Bodman spoke, and Mr. Drew Smythe replied.

The seventh meeting of the session was held in the Physiological Lecture Theatre of the University on Wednesday, 13th April, at 8.30 p.m.

Dr. MAYES showed skiagrams and Dr. FRASER pathological specimens.

The following resolutions were passed unanimously:—

1. That it is desirable to afford the friends and patients of the late Dr. Carey Coombs an opportunity to institute a suitable memorial to him.

2. With a view to carrying out this suggestion the following corporate bodies with whom Dr. Coombs was closely associated shall be asked to nominate two representatives each, to form with two representatives from this Society a memorial Committee.

The Bristol University.
The Royal College of Physicians.
The Bristol General Hospital.
The British Medical Association.

3. That the Society's representatives should be Mr. HEY GROVES and Dr. ELWIN HARRIS, junior.

Dr. FRANK BODMAN then read a paper on "**Common Nervous Disorders in General Practice.**"

The PRESIDENT complimented Dr. BODMAN on his paper, and a discussion followed in which Dr. NIXON, Dr. CARLETON, Dr. TODD, Dr. CASSON, Mr. GARDEN, Dr. CHARLES, Dr RUDOLPH and Dr. SAXBY spoke.

---

The last meeting of the session was held in the Physiological Lecture Theatre of the University on 10th May, at 8.30 p.m.

Dr. G. BUSH and Dr. A. L. TAYLOR showed and described skiagrams and pathological specimens respectively.

Mr. A. W. ADAMS read a paper on "**Gastric Surgery,**" which was discussed by the PRESIDENT, Mr. CLIFFORD MOORE, Dr. RICHARD CLARKE, Mr. CHITTY and Mr. E. WATSON-WILLIAMS and Mr. ADAMS replied.

Miss SYLVIA WIGODER then read a short paper on "**The Early Results of Radium Treatment in Operable Carcinoma.**"

This was discussed by the PRESIDENT, Mr. CLIFFORD MOORE, Dr. FARESLEY BROWN, Mr. E. WATSON-WILLIAMS, and Mr. DUNCAN WOOD.

Miss WIGODER replied.

---

### Bath and Bristol Surgical Club.

The Bath and Bristol Surgical Club met at the Hospital for Sick Children, Great Ormond Street, London, on 2nd June.

Mr. OSWALD ADDISON performed an operation for closure of cleft palate in a child of two.

Mr. BARRINGTON WARD removed the spleen for relief of acholuric jaundice in a boy of twelve.

Mr. TYRRELL GRAY performed Rammstedd's operation on two babies, and Bevan's operation on a boy with undescended left testicle.

A large collection of orthopædic cases and general skiagrams and specimens was demonstrated. The members subsequently dined together at the Dorchester Hotel.

---

### Visit of the Section of Surgery of the Royal Society of Medicine.

On Wednesday morning, 7th June, fourteen members of the Surgical Section of the Royal Society of Medicine came to the Bristol Royal Infirmary for the Surgical Meeting.

Dr. TODD gave a short discourse on "**The Chemotherapeutic Treatment of Cancer.**" Following this he demonstrated cases in which growths remained absent for three or four years after treatment, including among others :—

1. Sarcoma of the upper jaw.
2. Lympho-sarcoma of the left side of the neck.

3. Recurrent cancer of the rectum, overcome by radium treatment.

4. Cases of Hodgkin's disease in which the remaining glands were only very tiny ones.

Mr. C. F. WALTERS showed two patients on whom he had performed adrenalectomy.

Mr. H. CHITTY showed a girl with a scarred neck—the result of actinomycosis—which had been cured by tincture of iodine in milk. He reported that in fourteen cases only one had not been cured by this treatment. He then discussed the operation for Dupuytren's contracture.

Mr. W. A. JACKMAN showed a patient in whom only two digits were present on either hand. In addition he showed two cases of anomalous bone infections.

Professor RENDLE SHORT removed a carcinoma of the rectum by perineal excision, showed two patients on whom he had successfully operated for exophthalmos, and a case of ectopia vesicæ successfully treated by transplantation of the ureters and excision of the bladder.

Mr. A. WILFRID ADAMS showed operation specimens of tubercular and carbunculous kidneys and a stomach with four active ulcers along the lesser curvature.

Some of the members then visited the Ear, Nose and Throat Theatre to inspect the new operating table.

After lunch at the Infirmary the visitors went on to the General Hospital and were received by the Surgical Staff.

A number of cases of injury and disease of the spine were shown, including several compression fractures of the bodies of the vertebræ, treated by fixation in plaster in a position of hyper-extension; a girl of 20 who had a fracture dislocation of the lumbar vertebræ treated by open reposition, followed by a bone graft, with complete recovery. An interesting discussion took place on the relative advantages of fixation of compression fractures of the spine by plaster or by an immediate bone graft. Another case which provoked some discussion was that of a man of 50 who had a disease of the third lumbar vertebra, which had caused almost complete disappearance of the body. Probably this was a case of myeloma, and under the action of deep X-rays great improvement had taken place. The patient was on the new pattern of the reversible spinal bed, and the visitors showed great interest in the demonstration of his being turned over. Other cases shown were: a recurrent dislocation of the shoulder, treated by a fascial sling; a girl with a myeloma of the lower end of the femur, treated by excision and bone grafting; two infants with large cystic swellings of the buttock, probably teratomata.

Dr. ROGERS showed a number of recent specimens in the pathological museum.

